# What predicts instructional quality and commitments to teaching: self-efficacy, pedagogical knowledge or integration of the two?

**DOI:** 10.3389/fpsyg.2024.1287313

**Published:** 2024-01-29

**Authors:** Äli Leijen, Margus Pedaste, Aleksandar Baucal, Katrin Poom-Valickis, Liina Lepp

**Affiliations:** ^1^Institute of Education, University of Tartu, Tartu, Estonia; ^2^Institute of Psychology, University of Belgrade, Belgrade, Serbia; ^3^Institute of Education, Tallinn University, Tallinn, Estonia

**Keywords:** general pedagogical knowledge, self-efficacy, instructional quality, commitments to teaching, in-service teachers

## Abstract

The aim of the paper is to identify different groups of in-service teachers based on their general pedagogical knowledge (GPK) and self-efficacy beliefs and to explore potential differences among these groups regarding their instructional quality and commitments to teaching. A sample of 161 in-service subject teachers (science, mathematics, or Estonian language) who taught in lower secondary schools in Estonia were included in the study. Data was collected with a GPK test and self-reported questionnaires on instructional quality and commitments to teaching in the context of an OECD Teacher Knowledge Survey. Based on the cluster analysis, three groups of in-service teachers were identified: “the over-confident” teachers with average self-efficacy and very low GPK, “the competent” teachers with high self-efficacy and GPK, and “the insecure” teachers with low self-efficacy and average GPK. These three types of teachers were different in terms of instructional quality and commitments to teaching. It seemed that teachers’ self-efficacy beliefs are more important than GPK for instructional quality; however, GPK is more important for teachers’ professional persistence illuminating their general sense of professional identity. Implications of these findings for teacher education and teacher retention will be discussed.

## 1 Introduction

Quality of teaching is generally considered as one of the major attributions for students’ learning (Hattie and Yates, 2014). Although Hattie’s and his colleagues’ work on visible learning provides a comprehensive overview of effective educational practices, scholars from different contexts and disciplinary traditions are still scrutinizing the exact components, processes, and underlying elements of high-quality teaching (Cochran-Smith, 2003; Kunter et al., 2013; La Velle and Flores, 2018). For example, researchers have focused on personal qualities of good teachers (Goodlad, 1990), on specific teaching/learning practices (Gage, 1978), on teacher thinking and knowledge (Shulman, 1986), and on the motivational components of teaching (Alexander, 2008). While researchers have diverse foci in approaching teaching quality, teacher knowledge and motivational dispositions are most clearly empirically supported as the core component of the teaching quality (see, e.g., Kunter et al., 2013; Fauth et al., 2019; Blömeke et al., 2022).

Teacher knowledge and thinking have been the major research tradition of teacher education since the eighties (see Cochran-Smith, 2004 for a historical overview). It has been guided by the influential works of Lee Shulman (1986, 1987) on teacher knowledge. Traditionally, three types of teacher knowledge were identified: content knowledge (the knowledge of the subject), pedagogical content knowledge (the knowledge about teaching and learning a specific subject), and general pedagogical knowledge (pedagogical knowledge not linked to the subject matter; hereafter referred to as GPK) (Shulman, 1987; König et al., 2011). This multidimensional nature of teacher knowledge has been empirically confirmed (König et al., 2016). However, content knowledge and pedagogical content knowledge have received more attention in teacher knowledge and thinking studies during the last decades (see, e.g., Ball et al., 2008; Baumert et al., 2010). Empirical studies focusing on GPK, which is also the scope of the current study, are less common (König et al., 2011; Guerriero, 2017; Leijen et al., 2022a). At the same time, GPK is a powerful resource for effective learning and teaching and is associated with higher student learning outcomes (see, e.g., Ulferts, 2021).

In addition to studies related to teacher knowledge, studies focusing on teacher motivational dispositions have increased in parallel to the development of theories of motivation in the field of psychology (e.g., socio-cognitive theory, achievement goal theory, expectancy-value theory, and self-determination theory). For example, increasing attention is paid to teaching motives, self-and collective efficacy, and responsibility beliefs (see, e.g., Sonmark et al., 2017 for an overview). Although teachers’ knowledge base and motivational dispositions are argued to be related in theoretical studies (Sonmark et al., 2017) and also shown to be related in empirical studies (see, e.g., Kunter et al., 2013; Fauth et al., 2019), it is still unclear how the more specific aspects of the teacher knowledge base, such as codified knowledge of formal theory, interact with the implicit and automated knowledge and different motivational attributes, and also influence teaching practices (see, e.g., Grosemans et al., 2015). Therefore, it is essential to further analyse the interplay between teachers’ knowledge and motivational dispositions in order to better understand how to design learning opportunities for pre-and in-service teachers.

In this study, we explore the relationship between GPK and self-efficacy beliefs among teachers aiming to distinguish different groups of teachers based on these two broad characteristics as well as to explore how the different groups report their instructional quality and commitments to teaching as indicators of teachers’ work and discuss the implications of these findings for teacher education and teacher retention. Several countries are currently experiencing problems with teacher shortages and teacher retention, and some of the most important factors supporting teacher retention are high-quality preparation and professional development opportunities (Crehan, 2016; Podolski et al., 2016; Amitai and Van Houtte, 2022); this makes our research especially timely and relevant. Following, we will present the theoretical framework and research questions of the current study.

## 2 Teachers’ general pedagogical knowledge

Shulman (1987) proposed a framework for describing teachers’ knowledge. He initially distinguished seven categories: content knowledge, general pedagogical knowledge, curriculum knowledge, pedagogical content knowledge, knowledge of learners and their characteristics, knowledge of educational contexts, and knowledge of educational ends, purposes, and values. General pedagogical knowledge was, in this context, defined as “broad principles and strategies of classroom management and organization that appear to transcend subject matter” (Shulman, 1987, 8). Later, these seven categories have often been merged into three main categories – content knowledge, pedagogical content knowledge, and GPK. In this division, GPK also combines facets of curriculum knowledge, knowledge of learners and their characteristics, knowledge of educational contexts, and knowledge of educational ends, purposes, and values (see for example Baumert et al., 2010; König et al., 2016; Leijen et al., 2022a). In addition to that, previous work carried out by Voss et al. (2011) has considered GPK as a combination of pedagogical and psychological aspects, covering declarative knowledge of facts (knowing “that”) and procedural knowledge of skills (knowing “how”). In this paper, GPK definition relies on Guerriero (2017), 80 who has concluded that GPK is “the specialized knowledge of teachers in creating and facilitating effective teaching and learning environments for all students, independent of subject matter.” Based on this, GPK has been conceptualized into three main dimensions (instructional process, learning process, and assessment) that derived from previous work carried out with Teacher Education and Development Study in Mathematics test (TEDS-M; see for example König et al., 2011). Instructional process represents teachers’ knowledge that is necessary for planning and carrying out an effective and realistic lesson, while taking into account time management and the variety of teaching methods. Learning process dimension focuses on students’ development and the role of prior knowledge, information processing, and motivation in the learning process itself. Assessment dimension is considering the different ways to evaluate students, but also teachers’ ability to understand, interpret and apply information from research (König et al., 2011; Voss et al., 2011; Sonmark et al., 2017). Previous studies have demonstrated the relationship between teachers’ GPK, higher teaching quality, and better student results (Voss et al., 2011; Ulferts, 2021; Blömeke et al., 2022). Some previous studies have also identified qualitative differences regarding GPK among teachers (Klemenz and König, 2019; Nehls et al., 2020). For example, Nehls et al. (2020) identified two profiles of teachers based on their TEDS-M test results. The main variable distinguishing the profiles was knowledge of adaptivity (including knowledge about strategies of differentiation and the use of a wide range of teaching methods). Teachers with more knowledge of adaptivity showed significantly higher levels of cognitive action in class.

## 3 Teacher self-efficacy

Socio-cognitive theory developed by Bandura (1986) has been operationalized in studying self-efficacy beliefs. Bandura (1997) showed that efficacy beliefs are crucial in determining the effort people exert, their persistence in the face of challenges, their self-regulation and motivation, their accomplishments, and the decisions they make in life. Consequently, teacher self-efficacy refers to the beliefs teachers have regarding their confidence to execute their professional tasks. Efficacy beliefs are typically conceptualized as domain-specific and illuminate teachers’ confidence in carrying out particular activities. For example, Tschannen-Moran and Hoy (2001) distinguished between teachers’ perceived confidence in carrying out a variety of instructional strategies, perceived confidence in classroom management, and perceived confidence in influencing student engagement. Besides specific beliefs, some scholars have also investigated teachers’ general self-efficacy (see, e.g., Lauermann and König, 2016). Self-efficacy beliefs are positively related to teacher-reported and student-reported instructional practices (Thoonen et al., 2011). Lauermann and Berger (2021) indicate that teachers with higher self-efficacy tend to foster more student engagement and support student agency in learning. In addition, a meta-analysis of Klassen and Tze (2014) demonstrated positive relations between teacher self-efficacy and student achievement. Contrary, a more recent review by Lauermann and ten Hagen (2021) reviewed various meta-analyses and found that the correlation between teacher self-efficacy and student achievement is inconsistent, ranging from small positive to near-zero. This suggests that while a theoretical relationship is expected, it has been challenging to show it empirically. Moreover, it has also been found that a teacher’s self-efficacy has a positive relationship with factors underlying teachers’ psychological well-being (see Zee and Koomen, 2016, a synthesis of 40 years of research, for more details) and teacher-reported professional development aspirations (Klassen and Chiu, 2011).

Although, the above reported evidence regarding positive relations between efficacy beliefs and instructional practice would also suggest positive relationship between efficacy and GPK, Depaepe and König (2018) showed that while self-efficacy was related with instructional practice, no relationship was found between GPK and self-efficacy among pre-service teachers. Similarly, Dicke et al. (2015) found no significant relationship between teacher candidates’ educational knowledge and self-efficacy. Their study was conducted during the induction period of teacher education and lasted for 1 year. Contrary, Lauermann and König (2016) found a weak positive relation between GPK and self-efficacy among in-service teachers. Therefore, previous findings regarding the relationship between teachers’ GPK and self-efficacy are somewhat inconclusive and the dynamics between the two variables needs to be further studied.

## 4 Instructional quality

Instructional practice refers to the activities teachers carry out in the classroom to support their students’ learning. Klette et al. (2017) distinguish between four dimensions of instructional quality: instructional clarity (clear goals and instructions), cognitive activation (cognitive challenges and support), discourse features (quality of student-teacher interactions), and supporting climate (related to classroom management). Another tradition distinguishes between three dimensions which largely resonate with the above mentioned four: cognitive activation, classroom management, and social support for students (see, e.g., Kunter et al., 2008, 2013; Baumert et al., 2010; Depaepe and König, 2018). Cognitive activation relates to the extent to which teachers’ instructional strategies and the selected learning tasks are cognitively challenging for students. Classroom management deals with the efficient use of allocated classroom time, teachers’ expectations of student behavior, and the prevention of disorder in the classroom. Student support addresses issues of encouraging students and providing adaptive learner support. These three broad dimensions of instructional practice are related to students’ cognitive and non-cognitive learning outcomes (Baumert et al., 2010; Kunter et al., 2013; Fauth et al., 2019). Some studies (e.g., Gaytan and McEwen, 2007; OECD, 2014) have also distinguished between two means of cognitive activation: instructional strategies and assessment strategies.

Previous studies have found a positive relationship between teachers’ self-efficacy beliefs and their instructional quality. For example, Holzberger et al. (2013), 779 found in a longitudinal study that “teachers with higher self-efficacy beliefs reported higher cognitive activation, better classroom management, and more individual learning support for students.” It has also been shown that teachers with high self-efficacy in applying instructional strategies are more child-centered than those with low self-efficacy (de Laat and Watters, 1995).

Studies have also explored the relationship between instructional quality and GPK. For example König and Pflanzl (2016) and Lohse-Bossenz et al. (2015) showed that GPK is a significant predictor for instructional quality. In another study, teacher candidates, who were rated by pupils with high pedagogical and psychological knowledge scores were much more likely to provide cognitively activating learning environments, adapt the pace of their instruction to the needs of the students, relate to their students, recognize comprehensive problems, and prevent disruptions in the class (Voss et al., 2011). As indicated above, Nehls et al. (2020) identified qualitative differences regarding GPK among teachers and showed that teachers with more knowledge of adaptivity showed significantly more cognitive activation in class. However, unexpectedly, teachers did not differ regarding student support in relation to their knowledge of adaptivity. These findings suggest an overall positive relationship between GPK and instructional quality; however, qualitative differences regarding GPK in relation to different dimensions of instructional quality should be investigated in further studies.

## 5 Commitment to teaching

Commitment to teaching refers to a psychological bond or attachment to the teaching profession that expresses how teachers value and devote to the profession (Coladarci, 1992; Heinz, 2015; Leijen et al., 2022b). Sinclair (2008) summarizes that different levels of commitment can be described in several aspects: (1) in the choices of activities people do or avoid (termed “attraction”); (2) in the duration of their engagement in these activities (referred to as “retention”); and (3) in the level of intensity or depth of their involvement (described as “concentration”). Within the context of teaching and teacher education, these motivational factors are instrumental in determining what draws individuals to the teaching field, the length of time they stay in initial teacher education programs and later in the teaching profession, and the degree of their involvement and dedication to both their training and their professional roles. In line with the above, Lauermann et al. (2017) suggested investigating the following indicators of commitment, including “(1) commitment to teaching as a long-term career (inferred from planned persistence in teaching and career choice satisfaction), (2) interest in professional development as an indicator of professional engagement, and (3) willingness to invest personal time for teaching-related tasks (e.g., to help student learning)” (323). They argued that these indicators illuminate both the willingness to engage in professional activities and the commitment to teaching as a career (see also Watt and Richardson, 2008).

Research has shown that the degree of commitment to teaching is strongly related to self-efficacy (see, e.g., Coladarci, 1992; Hong, 2010; Klassen and Chiu, 2011; Chesnut and Burley, 2015). Klassen and Chiu (2011) explain that if teachers have both the necessary knowledge of teaching strategies and the ability to apply knowledge in practice, they are more committed to the teaching profession and more permanent in their job. Although there are no studies addressing directly the relationship between commitments to teaching and GPK, we expect based on the relationships between commitments to teaching and self-efficacy, the relation to be positive.

## 6 The present study

Previous research shows that GPK and self-efficacy are essential in both instructional quality and commitment to teaching. However, the findings regarding the relationship between teachers’ GPK and self-efficacy are somewhat contradicting, one study has shown positive relationship between the variables (Lauermann and König, 2016) while others have not (Dicke et al., 2015; Depaepe and König, 2018). These findings indicate that perhaps teachers are not a homogenous group with respect to the relationships between GKP and self-efficacy and it might be valuable to investigate qualitative differences between teachers’ groups regarding these two variables. For this reason we take a person-oriented approach (see, e.g., Bergman and Wångby, 2014) to identify different types of teachers with respect to these two variables in the current study:

1. Which groups of teachers can be distinguished based on their general pedagogical knowledge and self-efficacy beliefs?
2. To what extent are these groups of teachers different regarding their instructional quality and commitments to teaching?

Given our exploratory approach, we refrain from hypothesizing which groups are distinguishable and how these differ precisely regarding instructional quality and commitments to teaching. However, based on the previous findings reported above, we expect overall positive relationships between levels of GPK, self-efficacy, instructional quality, and commitments to teaching. Besides the possible scientific contribution furthering the understanding of the relationship between teachers’ GKP and self-efficacy, our study has high practical relevance for pre-and in-service teacher education since it might show which areas would need to be addressed in teacher education, and whether different groups of teachers would benefit from different content and pace for their professional development activities.

## 7 Methods

### 7.1 Sample

Data was collected in the context of an OECD Teacher Knowledge Survey (Sonmark et al., 2017). The current article is focusing on the data collected in Estonia, using convenience sampling. Out of all respondents, 78% filled in the full survey and only their data was included in the analysis. The final sample of the current study consists of 161 in-service teachers (82% women) from Estonia who teach science (57%), mathematics (26%) or Estonian language (21%) to children aged 13 to 15. Participants’ average age was 46.8 years (SD 12.7) and average teaching experience 21 years (SD 12.5). These characteristics of the sample are similar to the other representative samples (OECD, 2019) and the whole population of teachers in Estonia (according to the statistics of the Ministry of Education and Research^1^). Most of the in-service teachers worked full time (77%) and had finished teacher education at a higher education institute (89%). Data was collected anonymously. Participants gave informed consents for participating in the study, no incentives were provided for teachers for participation.

### 7.2 Measures

In this study, teachers’ GPK was measured with Teacher Knowledge Survey (TKS; Sonmark et al., 2017) based on a framework of three main dimensions of GPK as described in section 1.1 (instructional process, learning process, assessment). The final version of dataset used in this study included 50 test items from TKS. The detailed analysis of TKS psychometric properties on an Estonian dataset and sample items are presented in Malva et al. (2020) – reliability of item parameters was 0.99, and reliability of teacher GPK was 0.77. While the easiest items of the test were based on situational description, the most difficult items were related to theoretical concepts. This indicates that higher test scores would indicate a higher level of theoretical knowledge.

The self-report surveys about teachers’ self-efficacy, instructional quality and commitments to teaching were filled in before the knowledge test without time limit in an electronic environment (see Appendix). Teacher Self-Efficacy Scale (Tschannen-Moran and Hoy, 2001) measuring efficacy in student engagement, efficacy in instructional strategies and efficacy in classroom management was utilized to investigate teacher self-efficacy beliefs. In addition, a subscale of efficacy for student achievement (Lauermann and Karabenick, 2013) was also incorporated and the final survey instrument consisted of 15 items measuring teacher self-efficacy. CFA showed that the four factor model fits to data very well (*χ*^2^/df = 1.07, RMSEA = 0.021, CFI = 0.996, TLI = 0.994, SRMR = 0.045). It was also confirmed that these four factors can be presented as one higher order latent variable describing self-efficacy beliefs (*χ*^2^/df = 1.14, RMSEA = 0.029, CFI = 0.991, TLI = 0.989, SRMR = 0.053) and this measure was used in the following analysis.

Seven subscales were utilized to investigate instructional quality: social support, cognitive autonomy support, and monitoring subscales from Kunter et al. (2008), clarity of rules and teacher withitness subscales from Waldis et al. (2010), use of assessment (OECD, 2014), and differentiation subscale from Ramm et al. (2006). CFA showed that the seven factor model fits to data (*χ*^2^/df = 1.30, RMSEA = 0.043, CFI = 0.955, TLI = 0.946, SRMR = 0.060).

Commitments to teaching were investigated with the following subscales: planned persistence in teaching (Watt and Richardson, 2008), willingness to invest personal time (Lauermann et al., 2017), and interest in professional development (Lauermann et al., 2017). CFA showed that the three factor model fits to data moderately (*χ*^2^/df = 2.10, RMSEA = 0.083, CFI = 0.952, TLI = 0.933, SRMR = 0.055).

### 7.3 Analysis

Having in mind that the number of clusters was not known *a priori*, and that we have a relatively small sample of teachers, we have used the hierarchical cluster analysis (Ward method with squared Euclidian distance) for identification of relatively homogeneous groups of teachers based on GPK and self-efficacy scores. Before the variables related to GPK and self-efficacy were included in the cluster analysis, they were standardized because the original scales for GPK and self-efficacy variables were very different. Number of clusters was identified based on the analysis of the dendrogram presenting a nested sequence of clustering of teachers in different hierarchically organized clusters as well as scaled distances between clusters (Supplementary material). The key criteria for the selection of a specific number of clusters was the size of the scaled distance between two joining clusters.

The clusters of teachers, that were identified based on the hierarchical cluster analysis, were compared based on their standardized scores on instructional quality and commitments to teaching. Differences between the groups were analyzed with MANOVA tests with clusters as independent factor, and instructional quality and commitments to teaching dimensions as dependent variables. In all analyses participants with missing data as well as outliers (evaluated based on Mahalanobis Distances) have been excluded. Additional assumptions for MANOVA analysis are evaluated based on VIF statistics for absence of multicollinearity and Box’s M test for Equality of covariance matrices and they showed that preconditions for MANOVA analysis were met. The analysis has been done by SPSS ver. 25.

## 8 Results

Descriptive statistics of all measures are presented in Table 1. None of the constructs measured in the study reached a ceiling effect. Therefore, the test results from the Teacher Knowledge Test and about teacher self-efficacy, instructional quality, and commitments to teaching were further used in answering the research questions of the current study.

**Table 1:** Descriptive statistics of the measures used in the study (*n* = 161).

Variable	M	SD
Teacher knowledge test (max = 50)	28.46	5.68
Teacher self-efficacy (max = 7)	4.79	0.73
Instructional quality		
Social support (max = 4)	3.44	0.37
Cognitive autonomy support (max = 4)	2.52	0.63
Use of assessment (max = 4)	2.27	0.50
Monitoring (max = 4)	3.20	0.57
Clarity of rules (max = 4)	3.12	0.69
Teacher withitness (max = 4)	3.38	0.46
Differentiation (max = 4)	3.15	0.78
Commitments to teaching		
Planned persistence in teaching (max = 7)	5.65	1.20
Willingness to invest personal time (max = 7)	4.83	1.07
Interest in professional development (max = 7)	5.73	0.88

### 8.1 Groups of teachers based on self-efficacy and GPK

The analysis of the dendrogram presenting different hierarchical clustering of teachers based on the self-efficacy beliefs and GPK (see Supplementary material) suggested that the solution with three clusters is the optimal one compared to the two cluster solution and the solutions with more than 3 clusters.

**Figure 1:**
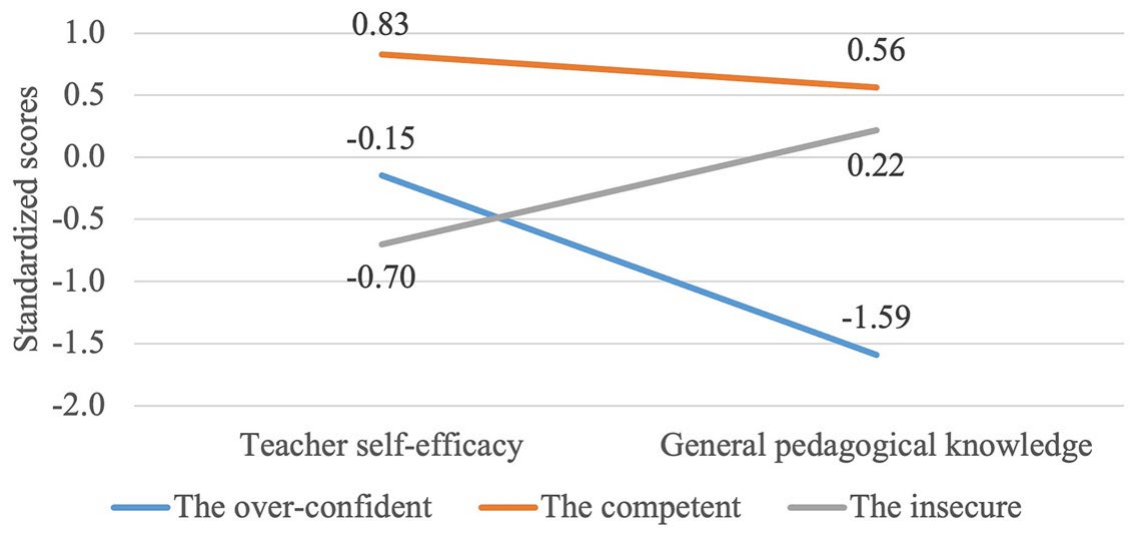
Three teachers’ groups composed based on self-efficacy and GPK.

Cluster analysis revealed three groups of teachers (see Figure 1). Group 1, which we named as “the over-confident” was composed of 27 teachers (18%) who were characterized by average scores of self-efficacy beliefs and very low scores on GPK. Group 2, which we named “the competent,” was composed of 60 teachers (39%) who were characterized by high scores of self-efficacy and high scores of GPK. Group 3, which we named “the insecure” was composed of 65 teachers (43%) who were characterized by low scores of self-efficacy and average scores of GPK. MANOVA analysis has shown that there are statistically significant differences between three clusters in terms of the teacher self-efficacy and GPK (*F* (4,296) = 101.52, *p* < 0.000; Wilk’s Λ = 0.178, partial *η*^2^ = 0.578). The ANOVA analysis for each of dependent variables also show that there are statistically significant differences between three clusters both in terms of teachers’ self-efficacy (*F* (2,149) = 70.46, *p* < 0.000, partial *η*^2^ = 0.486) and GPK (*F* (2,149) = 106.69, *p* < 0.000, partial *η*^2^ = 0.589). Finally, all pairwise comparisons between groups showed statistically significant differences (*p* < 0.01).

### 8.2 Differences between the groups regarding their instructional quality and commitments to teaching

**Figure 2:**
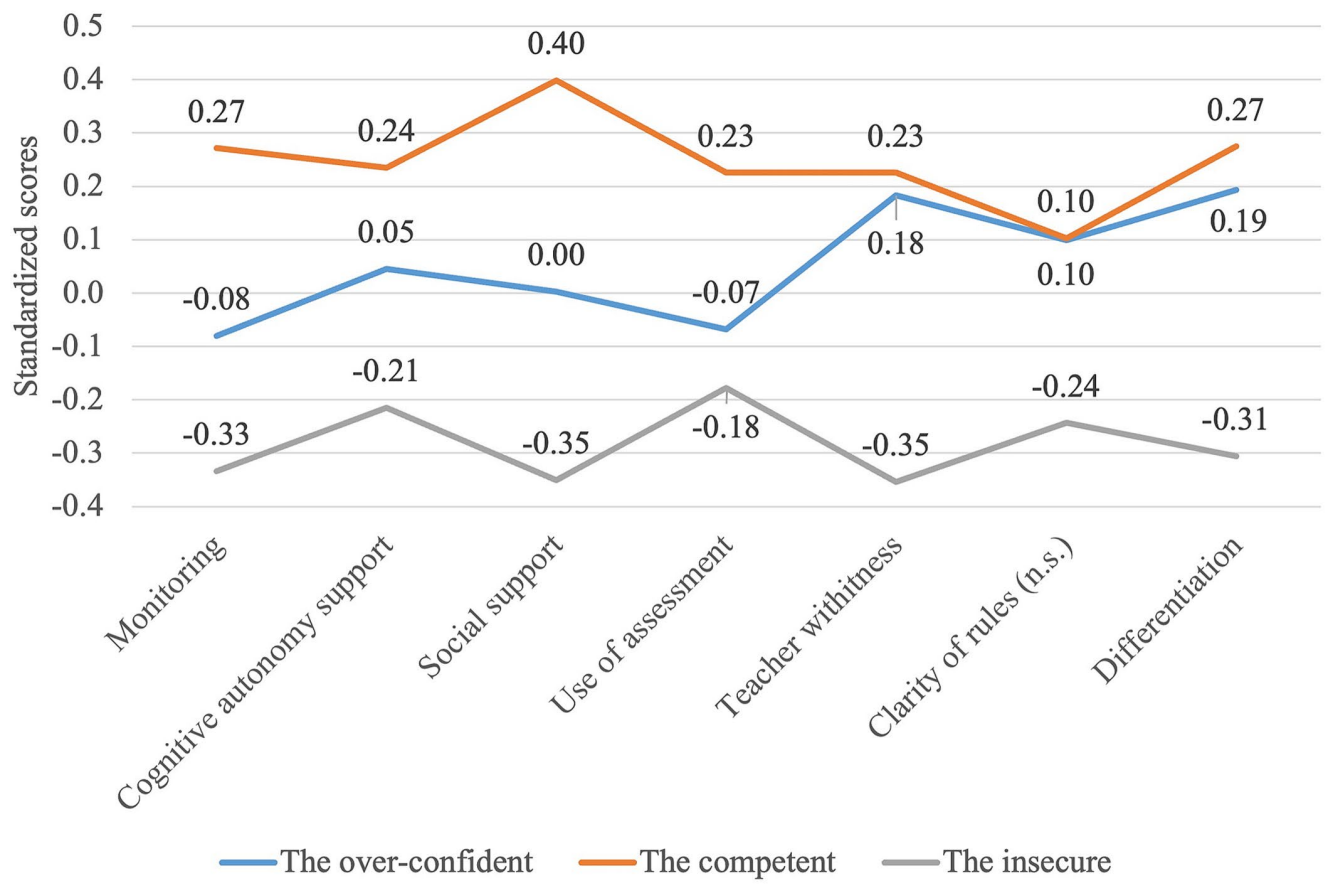
Differences regarding instructional quality between three teachers’ groups composed based on self-efficacy and general pedagogical knowledge.

The MANOVA analysis has shown that there are statistically significant differences between three clusters in terms of different dimensions of the instructional quality (*F* (14,272) = 2.345, *p* < 0.004; Wilk’s Λ = 0.796, partial *η*2 = 0.108). Furthermore, the univariate ANOVA analysis for each of the dependent variables showed that there were statistically significant differences between the groups of teachers in terms of 5 out of 7 dimensions of the instructional quality: social support, cognitive autonomy support, monitoring, teacher withitness, and differentiation (see Table 2 and Figure 2). Group 2, “the competent,” who had high self-efficacy and GPK scores reported also more frequent implementation of social support, cognitive autonomy support, monitoring of students and use of assessment than Group 3, “the insecure” (see Table 2). Interestingly, Group 1, “the over-confident,” who had average self-efficacy beliefs and very low GPK scores reported average level of implementation of social support, cognitive autonomy support, monitoring of students and use of assessment, and somewhat higher level of implementation of three classroom management aspects. Regarding these three aspects, this group answered very similarly to Group 2, “the competent,” who had high self-efficacy and GPK scores (more frequent reporting than Group 3, “the insecure”). Group 3, “the insecure,” who had low self-efficacy and average GPK scores reported lowest implementation of all dimensions of instructional quality. These findings suggest that low self-efficacy beliefs seem to be related to the lower frequency of implementation of different dimensions of instructional quality.

**Table 2:** Differences between three groups of teachers based on MANOVA.

	Average standardized score			F	Partial *η*^2^	*p*
Variable	Group 1, “the over-confident”	Group 2, “the competent”	Group 3, “the insecure”			
Instructional quality						
Social support	0.003	0.399	0.120	−0.350	9.652	<0.001
Cognitive autonomy support	0.045	0.235	0.042	−0.215	3.135	0.047
Use of assessment	−0.068	0.266	0.037	−0.177	2.701	0.071
Monitoring	−0.080	0.272	0.074	−0.334	5.470	0.004
Clarity of rules	0.099	0.103	0.028	−0.243	2.065	0.131
Teacher withitness	0.183	0.225	0.078	−0.354	5.966	0.003
Differentiation	0.193	0.275	0.075	−0.305	5.742	0.004
Commitments to teaching						
Planned persistence in teaching	−0.553	0.196	0.069	−0.005	5.488	0.005
Willingness to invest personal time	−0.072	0.242	0.039	−0.189	3.056	0.050
Interest in professional development	−0.366	0.420	0.116	−0.211	9,776	<0.001

**Figure 3:**
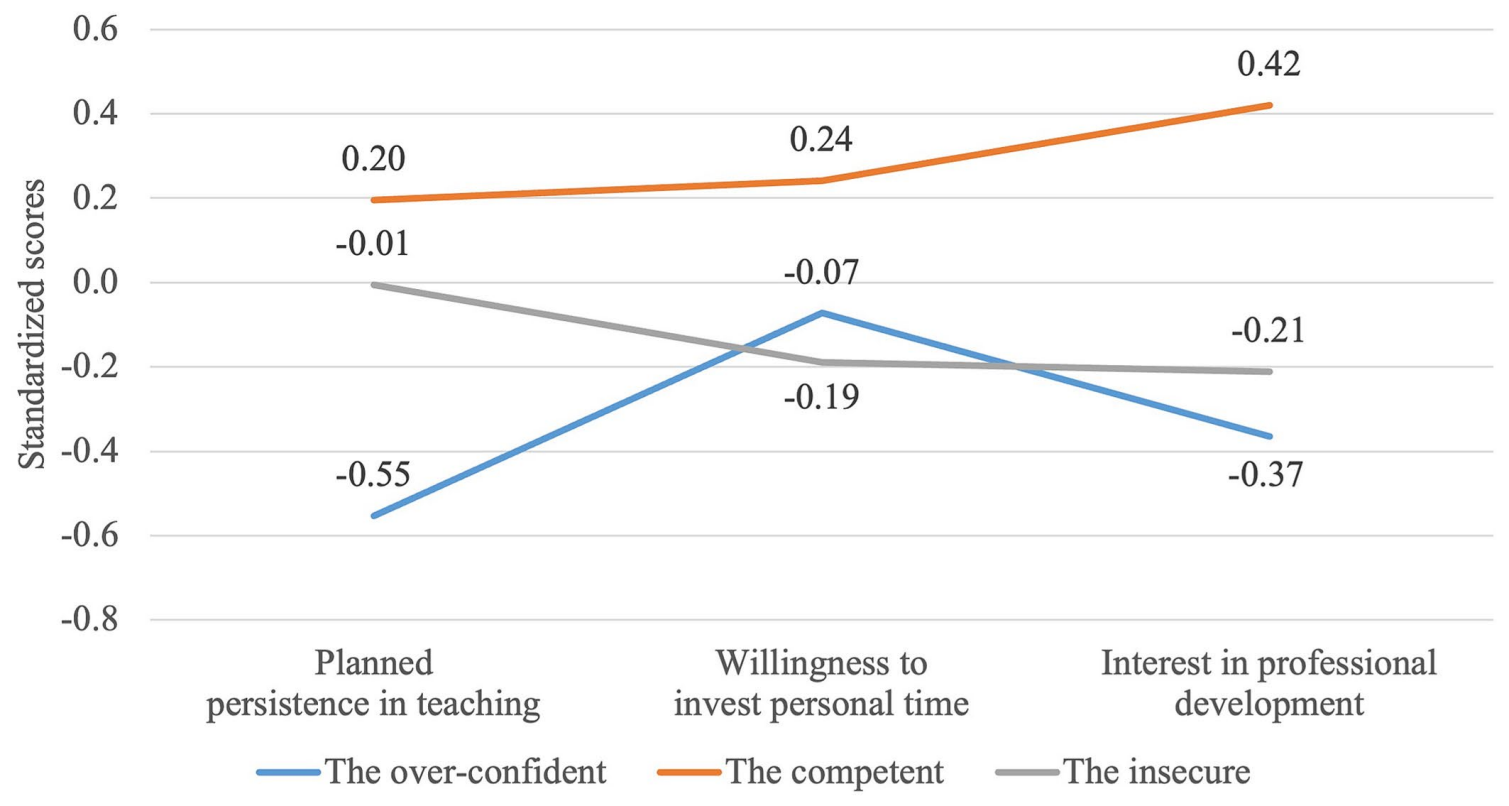
Differences regarding commitments to teaching between three groups of teachers composed based on self-efficacy and general pedagogical knowledge.

The MANOVA analysis has also shown that there are statistically significant differences between three groups regarding three dimensions of commitments to teaching – *F* (6,294) = 4.662, *p* < 0.000; Wilk’s Λ = 0.834, partial η2 = 0.087 (see Table 2 and Figure 3). Group 2, “the competent” who had high self-efficacy and GPK scores, reported higher agreement with professional persistence (higher than Group 1, “the over-confident”) and higher levels of willingness to invest personal time for professional tasks and interest in professional development (higher than both Group 1 “the over-confident” and 3 “the insecure”). Most interesting finding appeared regarding professional persistence in two other groups. On the one hand, Group 1, “the over-confident,” who had average self-efficacy and very low GPK scores, reported low agreement with professional persistence although, as we showed earlier, their reporting of instructional quality was partly similar to Group 2 “the competent.” On the other hand, Group 3, “the insecure,” who had low self-efficacy and average GPK scores, and who had previously reported lowest score in seven sub-scales of instructional quality, reported average agreement with professional persistence. Differences regarding willingness to invest personal time and interest in professional development were not statistically significant between Group 1 “the over-confident” and Group 3 “the insecure” (who had very different levels of GPK and self-efficacy beliefs). These findings suggest that GPK is related to teachers’ professional persistence.

## 9 Discussion

In this paper we distinguished three groups of teachers based on their GPK and self-efficacy beliefs and explored how the different groups reported their instructional quality and commitments to teaching. The instructional quality provided insight into the current teachers’ quality while commitment to teaching was important for providing insight into future perspectives of teachers and their interest to stay in teacher profession.

### 9.1 Teachers with highest GPK, self-efficacy, instructional quality and commitments to teaching

One of the groups of teachers showed higher levels of self-efficacy and GPK compared to the others. The same group reported higher levels on the most aspects of the instructional quality and commitments to teaching. These findings are in line with theoretical expectations (Shulman, 1987; Darling-Hammond and Bransford, 2005) and empirical findings from previous studies which have reported positive relationships between teachers’ GPK and instructional quality (Voss et al., 2011; Lohse-Bossenz et al., 2015; König and Kramer, 2016; König and Pflanzl, 2016). This relationship is probably mediated by another type of knowledge – pedagogical content knowledge (Lohse-Bossenz et al., 2013). The findings on commitments to teaching indicated that the most knowledgeable and motivated group of teachers was also most interested in professional development and most inclined to invest their personal time to teaching. This shows that these teachers are also very devoted to the profession and most likely in time their pedagogical expertise will only increase. It is also worth noting that in our study we found that about 39% of Estonian teachers participating in the study belong to this group that can be characterized as an ‘ideal’ group of teachers since they have high GPK, high level of self-efficacy and professional persistence, and they provide high quality instruction to students. In our view, it would be worth studying the professional trajectories of these teachers in order to understand how more teachers can be supported through initial teacher education and further professional development to become this kind of teachers.

### 9.2 Differences regarding teachers’ instructional quality

The most interesting finding regarding the instructional quality was the similarity of reporting on three subscales among teachers with high self-efficacy and GPK and among teachers with average self-efficacy and very low GPK. These three sub-scales: teacher withitness, clarity of rules, and differentiation are related to classroom management. The findings can therefore suggest that teachers’ self-efficacy beliefs seem to be more important than knowledge for several effective classroom management aspects. On the one hand, this finding is in line with previous studies on teachers’ self-efficacy. Goddard et al. (2004) have shown that the decisions teachers make about their classroom practices are directly influenced by their sense of efficacy for teaching. Similarly, mild uncertainty or strong doubts about one’s efficacy regarding the teaching of specific subjects, or use of specific teaching methods, can foster negative attitudes, interfere with teachers’ own learning and reduce use of new teaching approaches (Wheatley, 2002). These findings highlight the importance of opportunities to develop teacher self-efficacy during pre-and in-service teacher education.

On the other hand, this finding is also surprising, since previous studies have shown how theoretical educational knowledge predicts professional behavior (e.g., Lohse-Bossenz et al., 2015; König and Kramer, 2016), and more specifically, that knowledge on classroom management is related to classroom management practices (Voss et al., 2011). A previous study has shown that knowledge on classroom management should buffer against emotional exhaustion (Dicke et al., 2015), which in turn should protect instructional quality. However, the most difficult items of the GPK test were related to theoretical concepts, and higher test scores tended to indicate higher levels of theoretical knowledge. It is possible that in the current context, some aspects of instructional quality, namely classroom management, could be achieved with practical insights and without high levels of theoretical knowledge. Further research is needed to investigate this relationship with the GPK test that is more balanced with practical and theoretical items.

### 9.3 Differences regarding teachers’ commitments to teaching

Previous research (e.g., Klassen and Chiu, 2011; Zee and Koomen, 2016) has shown that higher levels of self-efficacy will protect teachers in times of difficulties and therefore support them in staying in their profession. Moreover, Lauermann and König (2016) showed that GPK negatively predicted teacher burnout both directly and indirectly via its positive association with teaching-specific self-efficacy. Somewhat contrary to these findings, our findings showed that rather theoretical GPK is more closely related to long-term commitment in the profession than self-efficacy. Teachers with very low GPK and average self-efficacy reported least satisfaction and willingness to persist in the profession. This is especially interesting in light with the findings regarding instructional quality, where the same group reported similar levels with the most knowledgeable teachers. These findings suggest differences between current practices and future perspectives. Teachers with high or average self-efficacy might compensate their low levels of theoretical knowledge base, e.g., GPK in a short term perspective; however, on the long term, it seems to be more difficult to persist in the teaching profession without a solid knowledge base as argued by several scholars (see, e.g., Gardner and Shulman, 2005). This finding has implications for teacher education. Teacher shortage is currently experienced in many countries and many alternative pathways to teaching have been designed. The alternative programs and pathways tend to emphasize practical experience and a quick introduction to the profession (see, e.g., critical review by Meijer, 2021). Although traditional teacher education programs are usually longer in comparison to the alternative approaches, these too are nowadays strongly focusing on practical preparation for teaching (Kelchtermans, 2017; Leijen and Pedaste, 2018). Moreover, because of the persistent issues of teacher shortages and retention, different stakeholders, such as policy makers, school owners and school leaders, are expecting traditional university programs to revisit their programs and to redesign these for more school-based, faster and more flexible alternatives (Estonian Ministry of Education and Research, 2021). Contrary to these expectations, the findings from the current study emphasize that ensuring a sufficient number of qualified teachers who are engaged professionally and committed to stay in the teaching profession also requires investing in the theoretical foundations of the profession as also noted by other scholars (Biesta et al., 2015; Kelchtermans, 2017). This also means that in-service teachers need opportunities to update their knowledge base as the field of education advances. Professional development activities focusing on collaborative teacher inquiry have shown positive results (Leijen et al., 2022c) in this regard.

### 9.4 Limitations and directions for future research

This data collection was part of piloting a larger international study, the data were collected using convenience sample. This fact opens up the question of generalizability of key findings of the study. For example, we found that 39% of sample could be characterized as an ‘ideal’ group of teachers. It is possible that this type of teachers felt more confident to participate in the study in comparison to others and they could be overrepresented in our sample. However, when looking at the background data of respondents, it fits with the average profile of Estonian in-service teachers which suggests that the results could be applied for a wider population of in-service teachers in Estonia. Moreover, instructional quality, self-efficacy, and commitments to teaching were investigated based on teachers’ self-reports. This may lead to concerns regarding validity. However, teachers’ self-reports are widely used and considered an economical and valid source for instructional quality (see, e.g., Baumert et al., 2010; Kunter et al., 2013), although direct observations of additional variables could meaningfully supplement teachers’ self-reports in future studies. Moreover, since self-efficacy beliefs are not necessarily uniform across the different types of tasks teachers perform and across different subject matter they teach (Bandura, 1997), more in-depth studies would be valuable to further specify the relationships between the different aspects of teachers’ self-efficacy and instructional practices.

Furthermore, findings suggest that self-efficacy, compared to the rather theoretical GPK, is more closely related to instructional quality. Considering that self-efficacy and instructional quality are measured by a self-report instrument while the GPK is measured by a knowledge test it opens up a question whether this finding reflects relationship between variables, or it reflects the way how variables were measured. However, based on the finding that the GPK proved to be associated with teachers’ commitments to teaching that was measured by a self-report instrument, we assume that our key findings are valid regardless of the difference in terms of operationalization of key constructs in our study.

## Data availability statement

The original contributions presented in the study are included in the article/Supplementary material, further inquiries can be directed to the corresponding author.

## Ethics statement

Ethical approval was not required for the study involving human participants in accordance with the local legislation and institutional requirements. The study was conducted in accordance with the local legislation and institutional requirements. The participants provided their informed consent to participate in this study.

## Author contributions

ÄL: Conceptualization, Data curation, Formal analysis, Funding acquisition, Investigation, Methodology, Project administration, Resources, Writing – original draft. MP: Formal analysis, Methodology, Visualization, Writing – original draft. AB: Formal analysis, Investigation, Methodology, Visualization, Writing – original draft. KP-V: Conceptualization, Data curation, Funding acquisition, Investigation, Project administration, Writing – original draft. LL: Writing – original draft.

## Footnotes

^1^ www.haridussilm.ee
