# Supplementary figures and images for: What predicts instructional quality and commitments to teaching: self-efficacy, pedagogical knowledge or integration of the two?
Source: Front Psychol. 2024 Jan 29;15:1287313. doi: 10.3389/fpsyg.2024.1287313 (PMC10860407; doi:10.3389/fpsyg.2024.1287313)

Supplementary material. Dendrogram of the clusters.

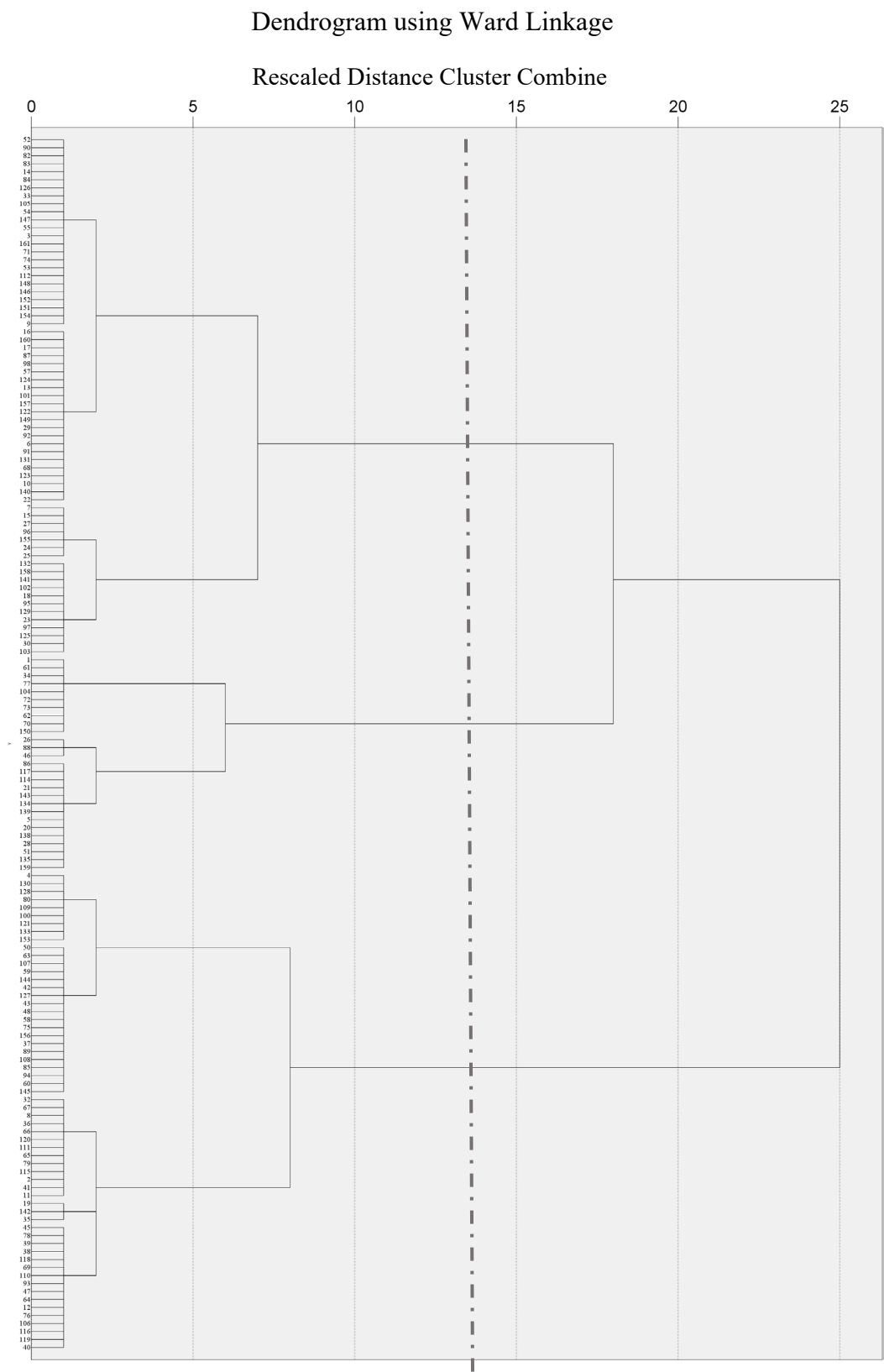

Supplement: Supplementary file 1 [file Image_1.pdf]
